# Association of Cross-Reactive Antibodies Targeting Peptidyl-Arginine Deiminase 3 and 4 with Rheumatoid Arthritis-Associated Interstitial Lung Disease

**DOI:** 10.1371/journal.pone.0098794

**Published:** 2014-06-05

**Authors:** Jon T. Giles, Erika Darrah, Sonye Danoff, Cheilonda Johnson, Felipe Andrade, Antony Rosen, Joan M. Bathon

**Affiliations:** 1 Division of Rheumatology, Columbia University, College of Physicians and Surgeons, New York, New York, United States of America; 2 Division of Rheumatology, Johns Hopkins University, Baltimore, Maryland, United States of America; 3 Division of Pulmonary and Critical Care Medicine, Johns Hopkins University, Baltimore, Maryland, United States of America; Keio University School of Medicine, Japan

## Abstract

**Background:**

A subset of rheumatoid arthritis (RA) patients have detectable antibodies directed against the peptidyl-arginine deiminase (PAD) enzyme isoforms 3 and 4. Anti-PAD3/4 cross-reactive antibodies (anti-PAD3/4XR) have been shown to lower the calcium threshold required for PAD4 activation, an effect potentially relevant to the pathogenesis of RA-associated interstitial lung disease (ILD).

**Methods:**

RA patients underwent multi-detector computed tomography (MDCT) of the chest with interpretation by a pulmonary radiologist for ILD features. A semi-quantitative ILD Score (range 0–32) was calculated. Concurrent serum samples were assessed for antibodies against PAD by immunoprecipitation with radiolabeled PAD3 and PAD4.

**Results:**

Among the 176 RA patients studied, any ILD was observed in 58 (33%) and anti-PAD3/4XR was detected in 19 (11%). The frequency of any ILD among those with anti-PAD3/4XR was 68% vs. 29% among those with no anti-PAD (crude OR = 5.39; p = 0.002) and vs. 27% among those with anti-PAD4 that was not cross-reactive with PAD3 (crude OR = 5.74; p = 0.001). Both associations were stronger after adjustment for relevant confounders (adjusted ORs = 7.22 and 6.61, respectively; both p-values<0.01). Among ever smokers with anti-PAD3/4XR, the adjusted frequency of any ILD was 93% vs. 17% for never smokers without the antibody (adjusted OR = 61.4; p = 0.001, p-value for the interaction of smoking with anti-PAD3/4XR<0.05).

**Conclusions:**

The prevalence and extent of ILD was markedly higher among RA patients with anti-PAD3/4 cross-reactive antibodies, even after accounting for relevant confounders, particularly among ever smokers. These findings may suggest etiopathologic mechanisms of RA-ILD, and their clinical utility for predicting ILD warrants additional study.

## Introduction

Clinically significant interstitial lung disease (ILD) is observed in 8–15% of individuals with rheumatoid arthritis (RA) and is a major source of morbidity and mortality [1]–[4]. Prognosis after the development of symptomatic RA-ILD is poor, with life expectancy averaging only 2.6 years [1]. Progressive decline in lung function, supplemental oxygen requirement, physical disability, secondary pulmonary hypertension with right heart failure, and need for lung transplantation are severe consequences [4], [5]. Subclinical RA-ILD is even more common, with radiographic ILD, as assessed using computed tomography (CT), observed in up to 50% of RA patients [6]–[8]. Although limited by the lack of clinical trials, there are, to date, no pharmacotherapies proven to be effective in altering the natural history of RA-ILD once symptoms have developed [9], and robust biomarkers for predicting those at risk for RA-ILD are lacking.

The pathogenesis of RA-ILD is poorly understood. One mechanistic possibility involves the presence and consequences of pulmonary citrullinated proteins. Citrullination is the post-translational modification of arginine residues to citrulline catalyzed by the peptidyl-arginine deiminase (PAD) enzymes [10]. Citrullination results in a net loss of charge and a more hydrophobic protein, with implications for protein folding and tertiary structure [11], [12]. Citrullinated proteins are present in lung tissue of patients with RA-ILD [13], idiopathic pulmonary fibrosis (IPF), and even broncho-alveolar lavage fluid from heavy smokers [14]. Repertoire expansion of antibodies against citrullinated protein antigens (ACPA) is associated with RA susceptibility [15], articular damage [16], and the presence and extent of radiographic RA-ILD [7], suggesting that citrullination of lung proteins and/or pathogenic ACPA may contribute to the pathogenesis of RA-ILD, perhaps via interfering with the normal functioning of targeted proteins or via pathologic antigen/antibody interactions.

PAD function is dependent on calcium, with maximal PAD function requiring 5–10 mM concentrations. This threshold is difficult to achieve *in vivo* and perhaps serves as a physiologic regulator of citrullination. Recently, we reported the discovery of an antibody targeting an epitope common to both PADs 3 and 4 that was highly specific for RA and was strongly associated with progression of radiographic erosions [17]. Adding purified IgG from patients with PAD3/4 cross-reactive antibodies (anti-PAD3/4XR) to PAD4 resulted in increased *in vitro* histone citrullination at physiologic concentrations of calcium, an effect not observed with the addition of anti-PAD4 that was not cross-reactive with PAD3. Accordingly, we hypothesized that RA patients with anti-PAD3/4XR would demonstrate a greater prevalence and extent of RA comorbidities in which tissue citrullination and/or pathogenic ACPA may play an etiopathologic role, such as RA-ILD.

## Methods

### Study Participants

Participants were enrolled in ESCAPE RA (Evaluation of Subclinical Cardiovascular disease And Predictors of Events in Rheumatoid Arthritis), a prospective cohort study investigating subclinical cardiovascular disease in RA described previously [18], [19]. Participants met 1987 RA classification criteria [20], had RA≥6 months from diagnosis, and were 45–84 years of age without known prior pre-specified cardiovascular events. All patients provided written informed consent prior to enrollment, and the study and consent procedures were approved by the Institutional Review Board (IRB) of the Johns Hopkins Hospital. Ongoing analyses were approved by the Columbia University Medical Center IRB. Enrollment occurred between October 2004 and May 2006.

### Outcomes

#### Pulmonary outcomes

As described previously [18], cardiac multi-detector row CT (MDCT) scans were obtained using standard methods [21] with 3 mm thickness on a Toshiba Aquilion 64 scanner. With cardiac MDCT, only lung parenchyma from the level of the carina to the lung bases was included. The validity of cardiac MDCT for pulmonary parenchymal disease has been evaluated, with correlation>90% compared to high-resolution CT [22]. Scans were assessable in 176 of the 195 enrolled participants (91%) by an expert pulmonary radiologist using a previously described standardized method [23] and blinded to clinical characteristics. Characteristics of the subgroup with assessable scans did not differ from those of the full cohort (data not shown). An ILD score (ILDS) was calculated based on the presence and extent of ILD features [i.e. ground glass opacification (GGO), reticulation (R), honeycombing (HC), and traction bronchiectasis (TB)] using a semi-quantitative scale (0 = none, 1 = 1–25%, 2 = 26–50%, 3 = 51–75%, 4 = 76–100%) with a maximum total score possible of 32. Intra-observer concordance for detecting no ILD was 90%, and 100% for ILDS≥3. Pulmonary function testing (PFT), consisting of spirometry and assessment of carbon monoxide diffusing capacity (DLCo), was performed according to American Thoracic Society guidelines [24] at the second study visit, occurring a mean ± standard deviation of 21±3 months post-baseline. Any restriction and impaired diffusion were both defined as≤79% of predicted for forced vital capacity (FVC) and DLCo, respectively.

#### Measurement of circulating anti-PAD3/4XR

Antibodies against PADs 3 and 4 were assessed as previously described [17]. Briefly, patient serum was added to [^35^S] Methionine labeled *in vitro* transcribed and translated PAD3 or PAD4. Radiolabeled immune complexes were incubated with Protein A beads and subsequently washed and boiled in SDS sample buffer. Radiography was used to visualize immunoprecipitated proteins separated by gel electrophoresis. Proteins were quantified using densitometry and levels normalized a standard sample with the high titer anti-PAD3/4XR antibodies. Seropositivity was defined at ≥0.01 of the normalized value, as none of the non-RA control samples (from healthy controls and patients with psoriatic arthritis) had levels above this cutpoint [17].

#### Other measures

Demographics, current and past smoking, and medical and RA disease history were assessed by patient self-report. Forty-four joints were examined for swelling and tenderness by a single trained assessor and RA disease activity calculated using the Disease Activity Score for 28 joints with CRP (DAS28-CRP) [25]. The Stanford Health Assessment Questionnaire (HAQ) [26] was used to assess disability. Current and past use of glucocorticoids, biologic and non-biologic disease modifying anti-rheumatic drugs (DMARDs) was queried by detailed examiner-administered questionnaires. Radiographs of the hands and feet were scored using the van der Heijde modification of the Sharp method (SHS) [27] by a single experienced reader blinded to patient characteristics.

#### Other laboratory assessments

C-reactive protein (CRP) was measured by nephelometry (Dade Behring Inc., Deerfield, IL). RF was assessed by ELISA, with seropositivity defined at or above a level of 40 units. Serum samples obtained concurrently with CT scanning were assessed for anti-CCP (CCP2) using a commercial kit. The presence of shared epitope alleles in exon 2 of HLA-DRB1 was determined as previously described [19].

### Statistical Methods

After exploring the distributions of all variables, group-wise differences in normally distributed continuous variables were compared using t-tests, in non-normally distributed continuous variables using the Kruskal-Wallis test, and in categorical variables using the chi-square goodness-of-fit or Fisher’s exact test, as appropriate. The associations of PAD antibody status with dichotomous pulmonary outcomes were explored using ordinary logistic regression, and for continuous pulmonary outcomes with linear regression, with variables transformed to normality as required. Multivariable (MV) regression models were constructed, with potential confounders included that were associated with the primary exposure variable in univariate analyses at the p<0.20 significance level, to allow for residual confounding. Non-contributory covariates were excluded using the Likelihood-Ratio Test for nested models or Akaike’s Information Criterion, as appropriate. Variance inflation factors were calculated to ensure that collinear covariates were not co-modeled. All calculations were performed using Intercooled Stata 12 (StataCorp, College Station, TX). A two-tailed α of 0.05 was used throughout.

## Results

Among the 176 RA participants with interpretable lung CT scans, 56 (32%) had antibodies against PAD4; however, only 19 (11%) had anti-PAD3/4XR. Participant characteristics according to antibody status are summarized in Table 1. Compared with individuals negative for anti-PAD, those with anti-PAD3/4XR were slightly older and less likely to be a current smoker, with trends to significance for both. Those with anti-PAD3/4XR had a median disease duration 13 years greater than those with no anti-PAD (p<0.001) and were significantly more likely to be seropositive for RF or CCP2 compared with both the groups with no anti-PAD and those with anti-PAD4 not cross-reactive with PAD3, although associations with the individual autoantibodies were not as strong. As previously published [17], those with anti-PAD3/4XR had a significantly higher total SHS score compared with the other groups. Anti-PAD3/4XR was not associated with RA disease activity measures or treatments.

**Table 1:** Patient Characteristics According to PAD3/4 Cross-Reactive Antibody Status.

Characteristic	Total (n = 176)	No anti-PAD (n = 120)	Anti-PAD4 only (n = 37)	Anti-PAD3/4XR (n = 19)	p-value*
Age, years	59±9	59±8	58±9	62±7	0.093
Male, n (%)	71 (40)	43 (36)	21 (57)	8 (42)	0.60
White, n (%)	152 (86)	101 (84)	35 (95)	16 (84)	0.99
Any college, n (%)	133 (76)	86 (72)	31 (84)	16 (84)	0.25
Ever smoking, n (%)	105 (60)	80 (67)	16 (43)	10 (53)	0.23
Current smoking, n (%)	20 (11)	19 (16)	2 (5)	0 (0)	0.075
Reported lung disease (n = 168), n (%)	27 (16)	22 (19)	4 (12)	1 (6)	0.30
RA duration, years	8 (4–17)	7 (4–12)	15 (7–23)	20 (11–28)	<0.001
RF or CCP2 seropositivity, n (%)	137 (78)	87 (73)	33 (89)	18 (95)	0.043
RF seropositivity, n (%)	114 (65)	73 (61)	26 (70)	15 (79)	0.20
CCP2 seropositivity, n (%)	122 (70)	76 (64)	31 (84)	15 (79)	0.30
Any shared epitope alleles, n (%)	122 (70)	79 (67)	30 (81)	14 (74)	0.79
DAS28-CRP	3.7 (2.9–4.4)	3.6 (2.9–4.3)	3.5 (2.8–4.5)	3.8 (3.3–4.3)	0.68
CRP, mg/L	2.4 (1.1–7.7)	2.1 (1.0–7.1)	3.5 (1.5–9.5)	2.0 (1.6–5.4)	0.65
IL-6, pg/mL	3.7 (1.8–7.8)	3.6 (1.7–8.1)	3.9 (1.8–6.3)	3.7 (2.4–9.0)	0.24
Total SHS	8 (1–37)	5 (0–19)	12 (2–43)	56 (14–132)	<0.001
Pain (100 mm VAS)	21 (9–41)	21 (8–40)	23 (10–41)	20 (5–47)	0.71
HAQ-DI (0–3)	0.63 (0.12–1.25)	0.63 (0.12–1.25)	0.75 (0.12–1.25)	0.75 (0–1.88)	0.73
Current prednisone, n (%)	67 (38)	45 (38)	13 (35)	9 (47)	0.41
Current non-biologic DMARDs, n (%)	150 (86)	100 (84)	34 (92)	16 (84)	0.99
Methotrexate, n (%)	114 (65)	79 (66)	22 (59)	13 (68)	0.83
Leflunomide, n (%)	19 (11)	11 (9)	5 (14)	3 (16)	0.41
Current biologic DMARDs, n (%)	81 (46)	55 (46)	17 (46)	10 (53)	0.63
TNF inhibitors, n (%)	78 (45)	52 (44)	17 (46)	10 (53)	0.62
Number of failed DMARDs	1 (0–2)	1 (0–2)	1 (0–3)	2 (0–2)	0.75

Values are mean ± standard deviation or median (interquartile range) unless otherwise noted.

*p-values are for the comparison of the no anti-PAD vs. anti-PAD3/4XR groups.

PAD = peptidyl-arginine deiminase; anti-PAD3/4XR = anti-PAD3/4 Cross-Reactive antibodies; RA = rheumatoid arthritis; RF = rheumatoid factor; CCP2 = 2^nd^ generation anti-cyclic citrullinated peptide antibody; DAS = disease activity score; CRP = C-reactive protein; IL = interleukin; SHS = Total modified Sharp-van der Heijde Score; HAQ = Health Assessment Questionnaire Disability Index; TNF = tumor necrosis factor; DMARD = disease modifying anti-rheumatic drug.

### Anti-PAD3/4XR was Associated with CT-ILD in Unadjusted Analyses

Pulmonary outcomes according to antibody status are summarized in Table 2. Any ILD was observed in 58 participants (33%), among whom 22 (38%) demonstrated a predominant radiographic ILD pattern of GGO and 36 (62%) demonstrated R/TB/HC. The median ILD score was 0, with a range of 0–10 units. The median emphysema score was also 0, and ranged from 0–6. Among the 158 patients with PFTs, 44 (28%) had any abnormality, with 30 (21%) demonstrating restriction or impaired diffusion. Among the 168 patients with assessment of respiratory symptoms, 69 (41%) reported any symptoms, with a median number of symptoms of 0 (range 0–4).

**Table 2:** Pulmonary Outcomes According to PAD3/4 Cross-Reactive Antibody Status.

Characteristic	Total (n = 176)	No anti-PAD(n = 120)	Anti-PAD4 only(n = 37)	Anti-PAD3/4XR(n = 19)	p-value*
Any ILD, n (%)	58 (33)	35 (29)	10 (27)	13 (68)	0.001
Any GGO, n (%)	22 (13)	11 (10)	6 (16)	5 (28)	0.047
Any R/TB/HC, n (%)	36 (22)	21 (19)	8 (22)	7 (39)	0.045
ILD Score, units (0–32)	0 (0–2; range 0–10)	0 (0–2; range 0–6)	0 (0–2; range 0–10)	2 (0–2; range 0–10)	0.020
Emphysema score, units (0–32)	0 (0–0; range 0–6)	0 (0–0; range 0–6)	0 (0–0; range 0–4)	0 (0–0; range 0–2)	0.77
Any PFT Abnormality (n = 158), n (%)	44 (28)	33 (29)	9 (29)	2 (13)	0.23
Any PFT Restriction or Impaired Diffusion, n (%)	30 (21)	21 (22)	8 (27)	1 (8)	0.46
Any respiratory symptoms (n = 168), n (%)	69 (41)	47 (40)	13 (38)	9 (53)	0.43
Number of respiratory symptoms	0 (0–1; range 0–4)	0 (0–1; range 0–4)	0 (0–2; range 0–4)	1 (0–1; range 0–3)	0.55

Values are median (interquartile range) unless otherwise noted.

*p-values are for the comparison of the no anti-PAD vs. anti-PAD3/4XR groups.

PAD = peptidyl-arginine deiminase; anti-PAD3/4XR = anti-PAD3/4 Cross-Reactive antibodies; RA = rheumatoid arthritis; ILD = interstitial lung disease; GGO = ground glass opacification; R/TB/HC = reticulation/traction bronchiectasis/honeycombing; PFT = pulmonary function testing; Impaired Diff = impaired diffusion.

For univariate comparisons of pulmonary outcomes according to antibody status, the prevalence of any ILD was more than double among those with anti-PAD3/4XR compared with the group with no anti-PAD and the group with anti-PAD4 that did not cross-react with PAD3 (both comparisons p<0.05). The prevalence of both GGO and R/TB/HC was also higher among those with anti-PAD3/4XR compared with the other antibody groups. However, anti-PAD3/4XR was not associated with PFT abnormalities, nor the presence or number of respiratory symptoms.

### RA Characteristics were Associated with Radiographic ILD

The associations of participant characteristics with the presence of any ILD features on CT (i.e. an ILD score>0) are summarized in Table 3. Those with any ILD features on CT tended to be older than those without ILD and were more likely to be male (both with trends to significance). Any ILD was strongly associated with both current and past smoking, but not with reported lung disease. Those with any ILD were significantly more likely to be seropositive for RF or CCP2 and had a higher median circulating IL-6 level. Trends to significance were noted for higher total SHS and HAQ among those with vs. without ILD. Those with ILD were also significantly more likely to be treated with prednisone and current biologics, most of which were TNF inhibitors. ILD was not associated with methotrexate or leflunomide use, or the number of failed DMARDs. As expected, those with ILD were significantly more likely to have PFT restriction or impaired diffusion and report respiratory symptoms compared with those without ILD.

**Table 3:** Patient Characteristics According to the Presence of CT-ILD Features.

Characteristic	No ILD (n = 118)	Any ILD (n = 58)	p-value
Age, years	58±8	61±9	0.071
Male, n (%)	43 (36)	29 (50)	0.085
White, n (%)	101 (86)	51 (88)	0.67
Any college, n (%)	87 (74)	46 (79)	0.42
Ever smoking, n (%)	62 (53)	44 (76)	0.003
Current smoking, n (%)	7 (6)	14 (24)	<0.001
Reported lung disease (n = 168), n (%)	19 (17)	8 (14)	0.66
RA duration, years	8 (4–16)	8 (5–19)	0.22
RF or CCP2 seropositivity, n (%)	86 (73)	52 (90)	0.011
RF seropositivity, n (%)	71 (60)	43 (75)	0.047
CCP2 seropositivity, n (%)	73 (62)	49 (86)	0.001
Any shared epitope alleles, n (%)	80 (68)	43 (75)	0.34
DAS28-CRP	3.5 (2.8–4.3)	3.8 (3.2–4.5)	0.12
CRP, mg/L	2.3 (1.0–7.2)	3.5 (1.2–9.3)	0.58
IL-6, pg/mL	3.0 (1.6–7.0)	4.5 (2.3–9.5)	0.029
Total SHS	6 (0–26)	12 (2–55)	0.074
Pain (100 mm VAS)	19 (10–40)	24 (8–47)	0.52
HAQ-DI (0–3)	0.62 (0.12–1.25)	0.75 (0.25–1.50)	0.083
Current prednisone, n (%)	37 (31)	30 (52)	0.009
Current non-biologic DMARDs, n (%)	103 (88)	47 (81)	0.21
Methotrexate, n (%)	81 (69)	33 (57)	0.13
Leflunomide, n (%)	11 (9)	8 (14)	0.37
Current biologic DMARDs, n (%)	48 (41)	34 (59)	0.028
TNF inhibitors, n (%)	46 (39)	33 (57)	0.028
Number of failed DMARDs	1 (0–2)	1 (0–2)	0.80
Any PFT Abnormality (n = 158), n (%)	22 (21)	22 (43)	0.003
Any PFT Restriction or Impaired Diffusion, n (%)	12 (13)	18 (41)	<0.001
Any respiratory symptoms (n = 168), n (%)	40 (36)	29 (52)	0.046
Number of respiratory symptoms	0 (0–1)	1 (0–2)	0.017

Values are mean ± standard deviation or median (interquartile range) unless otherwise noted.

RA = rheumatoid arthritis; RF = rheumatoid factor; CCP2 = 2^nd^ generation anti-cyclic citrullinated peptide antibody; DAS = disease activity score; CRP = C-reactive protein; IL = interleukin; SHS = Total modified Sharp-van der Heijde Score; HAQ = Health Assessment Questionnaire Disability Index; TNF = tumor necrosis factor; DMARD = disease modifying anti-rheumatic drug; ILD = interstitial lung disease; PFT = pulmonary function testing; Impaired Diff = impaired diffusion.

### Anti-PAD3/4XR Remained Associated with Any ILD After Adjustment

**Figure 1:**
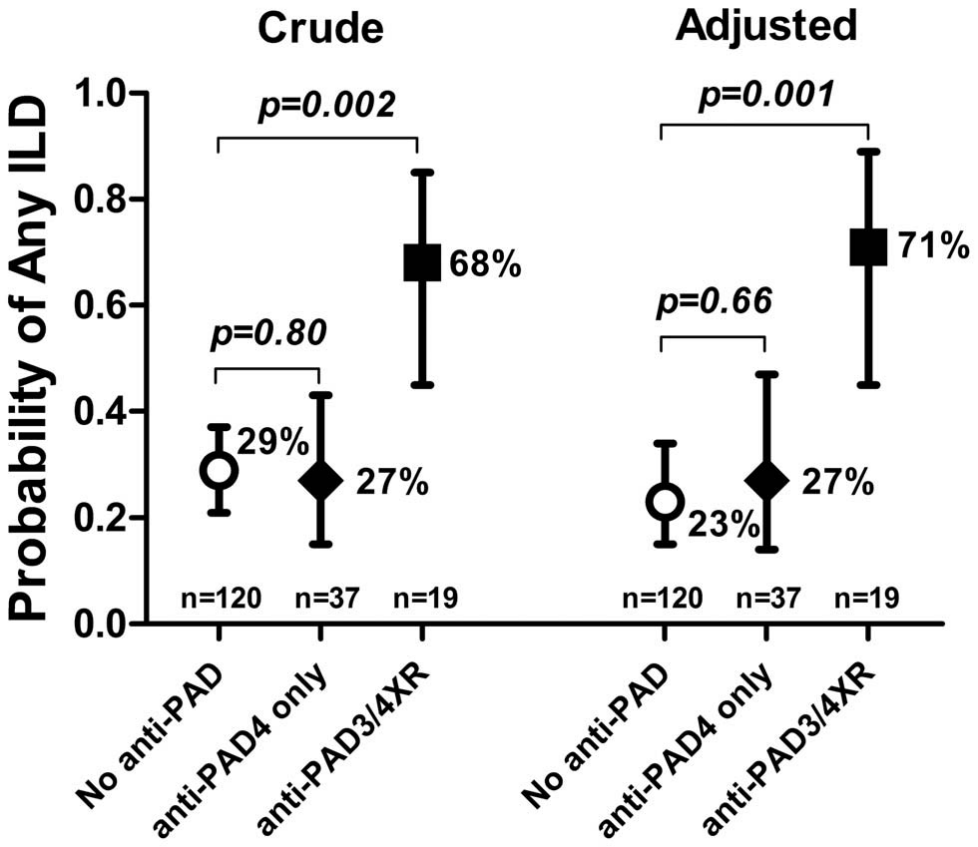
The Presence of anti-PAD3/4 Cross-reactive Antibodies was Associated with Radiographic RA-ILD. The cohort was grouped based on the presence of anti-PAD3/4XR antibodies (closed square), anti-PAD4 antibodies that did not cross-react with PAD3 (closed diamond), or neither reactivity (open circle). The prevalence of any ILD was more than double among those with anti-PAD3/4XR compared with those with anti-PAD4 alone or those with neither reactivity in both crude (left) and adjusted (right) analyses. Average probabilities and 95% confidence intervals are depicted. Associations adjusted for age, gender, current and past smoking, rheumatoid factor and CCP2 seropositivity, DAS28, current use of biologics and prednisone, RA duration, and total Sharp-van der Heijde Score.

Crude and adjusted associations of anti-PAD3/4XR with pulmonary outcomes are summarized in Figure 1. After adjusting for characteristics associated with either anti-PAD3/4XR or any ILD, anti-PAD3/4XR remained significantly associated with any-ILD compared with those with no anti-PAD or those with anti-PAD4 that did not cross-react with PAD3 (adjusted OR = 7.22 for the comparison of anti-PAD3/4XR vs. no anti-PAD; p = 0.001).

### The Combination of Anti-PAD3/4XR with Smoking was Highly Associated with Any ILD Features

**Figure 2:**
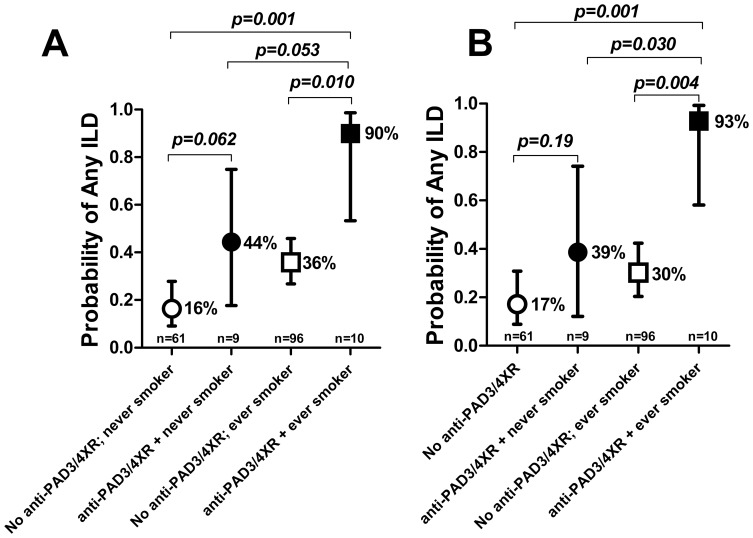
The Combined Effects of anti-PAD3/4 Cross-reactive Antibodies and Smoking Were Robust Indicators of Radiographic RA-ILD. The cohort was grouped based on the presence of anti-PAD3/4XR (closed markers) and history of ever smoking (i.e. current or former smokers). The association of anti-PAD3/4XR with ILD was stronger among ever smokers compared to never smokers in both crude (Panel A) and adjusted (Panel B) analyses. Average probabilities and 95% confidence intervals are depicted. Panel B associations adjusted for age, gender, rheumatoid factor and CCP2 seropositivity, DAS28, current use of methotrexate and prednisone, RA duration, and total Sharp-van der Heijde Score.

The individual and combined associations of anti-PAD3/4XR and ever smoking with any ILD are depicted in Figure 2. Before adjustment, only 16% of participants who were anti-PAD3/4XR negative and had never smoked had any ILD features on CT. The prevalence of any ILD was higher among those with either characteristic, and the magnitude of the association was similar for never smokers with anti-PAD3/4XR vs. ever smokers without the antibody. However, 90% of ever smokers seropositive for anti-PAD3/4XR demonstrated any features of ILD. The prevalence remained significantly higher for this group compared with the other groups after adjustment, resulting in an adjusted OR of 61.4 for the comparison of any ILD for ever smokers with anti-PAD3/4XR compared with never smokers without the antibody (p = 0.001).

### Adjusted Associations of PAD3/4XR with Other Pulmonary Outcomes

Associations of anti-PAD3/4XR with other pulmonary outcomes are summarized in Table 4. Both GGO and R/TB/HC were significantly higher among RA patients with anti-PAD3/4XR compared to those negative for the antibody in unadjusted analyses. However, only the predominant pattern of GGO was significantly associated after MV adjustment. Additionally after MV adjustment, anti-PAD3/4XR was associated with a higher ILD Score when considered as a continuous variable. However, anti-PAD3/4XR was not associated with any abnormalities on PFTs or the presence or number of reported respiratory symptoms in either crude or adjusted analyses.

**Table 4:** Crude and Adjusted Associations of PAD3/4 Cross-Reactive Antibodies with Pulmonary Outcomes in RA.

Outcome	Crude		Adjusted*		Adjusted**	
	β	p-value	β	p-value	β	p-value
Any ILD†	5.39	0.001	4.37	0.011	7.22	0.001
Any GGO†	2.99	0.042	3.93	0.034	3.50	0.043
Any R/TB/HC†	2.63	0.046	1.13	0.85	2.10	0.22
Any PFT Abnormality†	0.51	0.30	0.37	0.15	0.45	0.25
Any PFT Restriction or Impaired Diffusion†	0.48	0.36	0.40	0.26	0.59	0.52
Any respiratory symptoms†	1.81	0.22	1.74	0.29	2.15	0.17
Square root ILD Score	0.61	0.004	0.52	0.022	0.61	0.002

*Adjusted for age, RA duration, RF, CCP2, IL-6, SHS.

**Adjusted for age, gender, RF, CCP2, RA duration, DAS28, prednisone use, biologic DMARD use, current and past smoking, and SHS.

† β coefficients are Odds Ratios.

ILD = interstitial lung disease; GGO = ground glass opacification; R/TB/HC = reticulation/traction bronchiectasis/honeycombing; PFT = pulmonary function testing; Impaired Diff = impaired diffusion; RA = rheumatoid arthritis; RF = rheumatoid factor; CCP2 = 2^nd^ generation anti-cyclic citrullinated peptide antibody; DAS = disease activity score; CRP = C-reactive protein; IL = interleukin; SHS = Total modified Sharp-van der Heijde Score; DMARD = disease modifying anti-rheumatic drug.

## Discussion

In this first investigation of the relationship between the recently discovered RA-specific auto-antibody, anti-PAD3/4XR, and radiographic ILD, we detected a robust association for both the presence and extent of RA-ILD among patients with anti-PAD3/4XR compared with patients with no anti-PAD antibodies and even among those with anti-PAD4 antibodies that did not cross-react with PAD3. The associations were not diminished in magnitude under various relevant MV adjustment scenarios. Notably, the combination of anti-PAD3/4XR with a history of smoking was particularly robust in its association with RA-ILD, with a synergistic interaction suggested.

The etiopathology of RA-ILD is poorly understood. Adding to the complexity is heterogeneity in ILD histopathology and the high prevalence of asymptomatic disease [6]. However, in the setting of clinical symptoms the disease can be a severe and frequently lethal consequence of RA [1], [4]. Citrullination of lung proteins may contribute to the pathogenesis of RA-ILD. Fibroblast and lymphocyte migration through the pulmonary extracellular matrix to areas of damage, as well as cellular differentiation and activation, are dependent on interactions between matrix and cell surface proteins that are highly regulated [28]–[30]. Citrullination of key matrix proteins, therefore, could result in aberrant cellular migration, differentiation, and activation resulting in abnormal tissue repair, ectopic fibrosis, and dysregulated inflammation. For example, attachment and spreading of fibroblasts was impaired on plates coated with citrullinated fibronectin compared with non-citrullinated fibronectin [31]. This mechanism may not be exclusive to RA, as the presence of citrullinated proteins has been detected in the tissue targets of several autoimmune conditions, including the lungs of non-RA patients with ILD [13], [14]. An additional possibility is that citrullinated proteins in the lung are targets for ACPA, an interaction that may drive ILD. This possibility is supported by recent evidence for the presence of ACPA in the sputum of RA patients [32]. However, at present, any speculative mechanistic links between citrullinated proteins in the lung, ACPA generation and pathogenesis, and ILD remain to be elucidated.

While citrullination of lung proteins may contribute to the pathogenesis of ILD, physiologic barriers limiting PAD activity may have evolved to regulate such pathologic citrullination. For one, the calcium threshold for PAD catalytic activity generally exceeds levels attainable extra-cellularly. *In vitro* experiments typically use 5–10 mM calcium concentrations for maximal PAD activity, whereas *in vivo* extra-cellular levels typically do not exceed 1.5 mM [33]. Recently [17], we showed that anti-PAD3/4XR antibodies increased the sensitivity of PAD4 to calcium allowing efficient citrullination in the context of physiologic calcium concentrations. From the same report, anti-PAD3/4XR reacted with PAD4 at a key calcium binding site that is also involved in protein-protein interactions, suggesting that the antibody may create a permissive environment for calcium activated catalysis. Therefore, the analyses presented here provide a circumstantial link between a disease manifestation in which aberrant citrullination may play a pathogenic role (i.e. ILD) and the mechanism by which citrullination may be facilitated (i.e. anti-PAD3/4XR).

Smoking is a well-established risk factor for both RA [34] and ILD [35], and, as confirmed among the cohort reported here, RA patients with a history of smoking were more likely to have radiographic ILD. Additionally, compared with non-smokers, smokers had higher levels of PAD2 in the bronchial mucosa, but no difference in the amount of protein citrullination in the same tissue [14]. In our study, the combined effect of smoking plus anti-PAD3/4XR was greater than the sum of the individual effects of either characteristic in isolation, suggesting a possible synergistic interaction. In this setting, PAD up-regulation conferred by smoking combined with the enhanced catalytic activity of PAD4 conferred by anti-PAD3/4XR could provide the stimulus for the enhanced pathogenic effect observed. Based on this circumstantial evidence, testing of this speculative mechanistic hypothesis is warranted.

The study has notable strengths and limitations. Among the strengths, scans were interpreted by the same pulmonary radiologist with decades of ILD experience, and ILD features correlated with both PFT abnormalities and respiratory symptoms. Among the limitations, cardiac MDCT differs from high-resolution CT (HRCT) in slice thickness and the lung apices were not imaged. However, while we may have missed additional ILD features exclusive to the apices (uncommon for RA-ILD), our findings are internally consistent since the same techniques were used for all patients. Owing to the characteristics of the cohort, our findings may only be generalizable to RA patients >45 years of age without prior cardiovascular events, and, notably, there were few patients with severe RA-ILD included. Additionally, the primary comparisons were cross-sectional, limiting any ability to establish temporality in the associations. Additional longitudinal work is underway exploring the temporal relationship between emergence of anti-PAD3/4XR over the time-course of RA and incident RA-ILD.

In summary, anti-PAD3/4XR seropositivity was detected in a subset of RA patients with longer standing, seropositive, destructive disease. Independent of these features, the presence of anti-PAD3/4XR remained highly associated with the presence and extent of ILD, assessed by CT scanning. Interestingly, the combination of smoking history with anti-PAD3/4 antibody status was associated with a frequency of ILD to a greater extent than expected from the mere addition of their separate effects, suggesting a synergistic interaction. With additional investigation, these novel findings have potential implications for both understanding the mechanisms underlying the pathogenesis of RA-ILD and for predicting which RA patients are at risk for developing this potentially life-threatening RA extra-articular manifestation.
